# A real-world analysis of adherence, biochemical outcomes, and healthcare costs in patients treated with rosuvastatin/ezetimibe as single-pill combination vs. free combination in Italy

**DOI:** 10.1093/ehjopen/oeae074

**Published:** 2024-08-28

**Authors:** Alberto Zambon, Evangelos Liberopoulos, Melania Dovizio, Chiara Veronesi, Luca Degli Esposti, Leopoldo Pérez de Isla

**Affiliations:** Department of Medicine, University of Padua Medical School, 35128 Padova, Italy; 1st Department of Propedeutic Medicine, School of Medicine, National and Kapodistrian University of Athens, 15772 Athens, Greece; CliCon S.r.l., Società Benefit-Health Economics & Outcomes Research, 40138 Bologna, Italy; CliCon S.r.l., Società Benefit-Health Economics & Outcomes Research, 40138 Bologna, Italy; CliCon S.r.l., Società Benefit-Health Economics & Outcomes Research, 40138 Bologna, Italy; Cardiology Department, Hospital Clínico San Carlos, 28040 Madrid, Spain

**Keywords:** Adherence, Ezetimibe, Hypercholesterolaemia, Real-world evidence, Rosuvastatin, Single-pill combination

## Abstract

**Aims:**

To compare medication adherence, lipid goal attainment, and healthcare costs between patients receiving a single-pill combination (SPC) vs. a free combination treatment (FCT) of rosuvastatin/ezetimibe (ROS/EZE) in Italy.

**Methods and results:**

Administrative databases of healthcare entities covering ∼7 million individuals were used to identify adults prescribed with ROS/EZE as SPC or FCT between January 2018 and June 2020. Adherence was calculated as the proportion of days covered (PDC) after cohort balancing by propensity score matching. Patients with available LDL cholesterol testing were assessed for the proportion of those who at baseline were above lipid targets recommended by ESC/EAS Guidelines for their cardiovascular risk category and reached the target during follow-up. Among 25 886 patients on SPC and 7309 on FCT, adherent patients were more represented in SPC than FCT cohort (56.8 vs. 44.5%, *P* < 0.001), and this difference remained significant (*P* < 0.001) after stratification by cardiovascular risk (very high, high, and other). The proportion of patients reaching LDL cholesterol target at 1 year follow-up was significantly (*P* < 0.001) higher in SPC vs. FCT cohort: 35.4 vs. 23.8% for very high cardiovascular risk, 46.9 vs. 23.1% for high risk and 71.6 vs. 49.5% for other risk. Total healthcare costs per patient at 1 year follow-up were lower in SPC vs. FCT users (2337€ vs. 1890€, *P* < 0.001). In both cohorts, costs were mainly driven by drug expenses and hospitalizations.

**Conclusion:**

This real-world analysis in dyslipidaemic patients found that treatment with ROS/EZE as SPC resulted in better adherence, higher chances of reaching lipid goals, and cost savings over FCT, in all cardiovascular risk categories.

## Graphical abstract

**Graphical Abstract:**
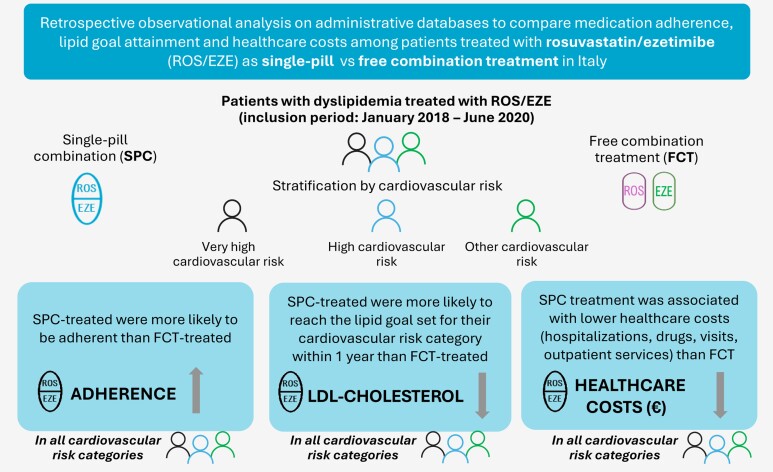

## Introduction

High circulating levels of LDL cholesterol (LDL-C) are a well-recognized causal risk factor for atherosclerotic cardiovascular disease,^1,2^ and LDL-C control through lipid-lowering therapy represents a major milestone of cardiovascular prevention.^3,4^ The latest guidelines of the European Societies of Cardiology and Atherosclerosis (ESC/EAS) have further lowered the bar for the target LDL-C levels to be reached, especially in patients at high and very high cardiovascular risk.^5^ However, many studies have shown an alarming gap between LDL-C values observed after lipid-lowering therapy and the targets recommended by the guidelines.^6–9^

One main barrier in achieving the lipid goals might lie in the risk of side effects for the liver and muscle with increasing statin dose^10^ that might lead to statin down-titration or discontinuation.^11^ Anyhow, some patients fail achieving LDL-C goals, even if treated with maximal doses of a statin.^11^

Several studies have described successful lipid-lowering effects when adding ezetimibe to statin monotherapy.^12–15^ Moreover, evidence from clinical trials showed that fixed-dose combinations of statins plus ezetimibe provided significantly superior benefits over statin alone to ameliorate overall lipid profile, by decreasing LDL-C, total cholesterol, and triglyceride levels.^15^

These positive results obtained with fixed-dose formulations of statins and ezetimibe corroborate the view that simplifying treatment regimens for patients might be helpful in improving adherence, which remains one major issue for successful achievement of lipid target.^5,16,17^

The reduction in the number of daily tablets has been proven to be an effective strategy to reach blood pressure goals with antihypertensive therapy in a recent analysis by Borghi *et al.*^18^ who reported that patients taking a combination of three drugs in a single pill were more adherent than those taking the same medications separately. Nevertheless, while the benefits of single-pill combination (SPC) on adherence have been largely described for antihypertensives,^18–22^ similar evidence still needs to be confirmed for lipid-lowering agents, as the likelihood of improved adherence using an SPC of statin and ezetimibe is still debated.^23–25^ Some discrepancies indeed have emerged in the current literature: while some studies described a better adherence in the users of SPC with fixed doses of simvastatin plus ezetimibe, simvastatin plus niacin, or lovastatin plus niacin over free combination treatment (FCT) regimens with separate pills,^24^ other reports failed finding such benefit.^25^

In Italy, data from the Lombardy region showed markedly better adherence to lipid-lowering therapy in patients prescribed with SPC of statin/ezetimibe than those who received two separate pills.^26^ A recent real-world analysis by our research group conducted on a representative sample accounting for nearly 12% of the Italian population revealed that patients treated with rosuvastatin and ezetimibe (ROS/EZE) who switched from FCT to SPC increased their odds of being adherent to lipid-lowering therapy.^27^

Although comparative analyses on the economic burden associated with the use of lipid-lowering agents as SPC or FCT are scanty, real-world evidence from claims of patients treated with drugs for cardiovascular diseases suggested the single-pill option to be associated with lower numbers of general practitioner (GP) and specialist visits, all-cause hospitalization days, and other drug prescriptions, resulting in cost savings compared with FCT.^28^

This real-world analysis was undertaken to evaluate and compare medication adherence, the likelihood of attainment of the recommended LDL-C targets, and direct healthcare costs between two cohorts of patients treated with rosuvastatin and ezetimibe (ROS/EZE) as SPC or FCT, in a representative sample of the Italian population.

## Methods

### Data source and administrative databases

A retrospective observational analysis was conducted using administrative databases of a sample of Italian Local Health Units (LHUs) covering ∼7 million health-assisted individuals and with data available from January 2017 to July 2021. Specifically, administrative flows contain all data regarding the healthcare resources/services dispensed and reimbursed the Italian National Healthcare System (INHS), stored in the following databases: beneficiaries’ database collecting patients’ demographics; pharmaceutical database containing data on all drug prescriptions with their Anatomical Therapeutic Chemical (ATC) code; hospitalization database including the hospitalization discharge diagnosis codes classified according to the International Classification of Diseases, Ninth Revision, Clinical Modification (ICD-9-CM); outpatient specialist service database for all data on specialist visits or diagnostic/laboratory tests, and laboratory test data of lipid profile (LDL-C values, available for a subset of LHUs).

Patients’ privacy was guaranteed, and a univocal numerical code was assigned to each subject included in the analysis, in full compliance with the European General Data Protection Regulation (GDPR) (2016/679). This code allowed the electronic linkage between the different repositories mentioned above. All the results of the analyses were produced and presented as aggregated summaries, so that it is impossible to identify, either directly or indirectly, individual patients. Informed consent was not required (pronouncement of the Data Privacy Guarantor Authority, General Authorization for personal data treatment for scientific research purposes—n.9/2014). The study was approved by all the ethics committees of the participating LHUs.

### Study design and population selection criteria

During the inclusion period, comprised between January 2018 and June 2020, all adults prescribed with ROS/EZE as SPC (ATC code C10BA06) or FCT (ATC codes C10AA07 for ROS and C10AX09 for EZE) were identified. The date of inclusion (index date) was that of the first prescription of SPC of ROS/EZE or that of the first prescription of ROS and EZE (with a maximum interval of 30 days between the prescriptions of each), during the inclusion period. The characterization period was defined as the whole period of data availability (at least 1 year) before the index date, and the follow-up was all available period (at least 1 year) after the index date.

### Baseline patient characteristics

At index date, demographic characteristics, namely age at inclusion and gender distribution expressed as percentage of male subjects, were collected. Comorbidity profile and medications were investigated during the characterization period. Specifically, among comorbidities, the frequency of previous chronic obstructive pulmonary disease (COPD, identified by at least two prescriptions for ATC R03), ischaemic heart disease (identified by ICD-9-CM codes 410–414), heart failure (ICD-9-CM code 428), cerebrovascular diseases (ICD-9-CM codes 430–438), peripheral vascular diseases (ICD-9-CM codes 440–442), chronic kidney disease (CKD, ICD-9-CM codes 585.3–5), diabetes mellitus (ICD-9-CM code 250 or at least two prescriptions of anti-diabetic drugs, ATC code A10), and psychiatric disease (ICD-9-CM codes 290–319) was assessed. The following medications (at least two prescriptions) were evaluated during the characterization period: statins other than rosuvastatin (ATC C10AA), antihypertensive treatments (ATC C03, C07, C08, C09), antithrombotic agents (ATC B01), antiarrhythmics (ATC C01B), anti-inflammatory and antirheumatic products (ATC M01), and antidepressants (ATC N06A). At baseline, patients were stratified by cardiovascular risk level in very high, high, and other (low or mild/moderate) cardiovascular risk. The stratification was based on the definition of cardiovascular risk level defined by the 2019 ESC/EAS Guidelines for the management of dyslipidaemias^5^ and adapted for the administrative database used (see Supplementary material online). Cardiovascular risk level was determined during the characterization period preceding the index date (specifically, any pharmacological treatments were evaluated in the 12-month period prior the index date, while hospitalization diagnoses during all the available period before the index date).

#### Evaluation of biochemical outcome

Patients not achieving ESC/EAS Guideline-recommended LDL-C target levels^5^ (below 55 mg/dL for very high cardiovascular risk, 70 mg/dL for high risk, and 116 mg/dL for other risk) at baseline and who achieved the lipid target after the first year of follow-up were identified in a subset of patients with at least one LDL-C measurement at baseline and during follow-up. Data were reported as the percentage of patients achieving the LDL-C target at 1-year follow-up over patients not achieving this target.

#### Propensity score matching analysis

The two cohorts of users of SPC and FCT of ROS/EZE were matched by applying the propensity score matching (PSM) to balance possible confounding variables using a 1:3 ratio. In particular, the following covariates were considered for matching: age at index date, gender, CKD, COPD, psychiatric disease, anti-inflammatory and antirheumatic treatments, antidepressant use, and cardiovascular risk level. The standardized mean difference (SMD) was used to compare the balance of the variables between the two cohorts. Standardized mean difference values above 0.2 were considered small, SMD values above 0.5 medium-sized, and SMD values above 0.8 large, as previously described.^29^

#### Evaluation of treatment adherence

Treatment adherence was compared between PSM-matched cohorts. Adherence to SPC was calculated as the number of days covered by SPC (assuming the consumption of 1 pill daily) during 12 months of follow-up. Adherence to FCT was calculated as the number of days covered by the two medications (assuming the consumption of two pills—one for each drug—daily) during 12 months of follow-up. The following cut-offs were used to stratify patients by adherence level: PDC < 25% (no adherence); PDC = 25–75% (partial adherence); PDC > 75% (adherence). Moreover, the percentage of adherent patients stratified by the level of cardiovascular risk was calculated.

#### Healthcare costs

The calculation of the mean direct healthcare costs per patient was based on drug prescriptions (all cause and cardiovascular-related), hospitalizations (all cause and cardiovascular-related), and outpatient services at 1-year follow-up, in the users of ROS/EZE as SPC or as FCT. Drug prices were computed using the INHS purchase price, hospitalization costs were determined using DRG (diagnosis-related group) tariffs, and costs of instrumental and laboratory tests were evaluated according to tariffs applied by each region. The determinants of increased or decreased healthcare annual direct costs during follow-up were also evaluated by a multivariable analysis.

#### Statistical analysis

Continuous variables are reported as the mean ± SD, whereas categorical variables are expressed as frequencies and percentages. For comparative analyses of percentages, χ^2^ test was applied. The SMD was used to compare the balance of the variables between the PSM-balanced groups of patients treated with SPC or FCT. Standardized mean difference (SMD) values above 0.2 indicated the unbalance between the groups.

Moreover, a generalized linear model was developed to identify the predictors of annual healthcare costs, checking for confounding factors such as age, sex, comorbidities, treatments, and cardiovascular risk categories, drug formulation (SPC or FCT), and adherence. A *P*-value of <0.05 was considered for statistical significance and all the analyses were performed using Stata SE version 17.0 (StataCorp, College Station, TX, USA).

## Results

Overall, 25 886 patients on SPC and 7309 patients on FCT were identified and included in the analysis. As shown in *Table 1*, the mean age at inclusion was 65.4 ± 11.0 and 65.7 ±11.0 years (*P* < 0.05), with 56.0 and 58.6% (*P* < 0.001) men among the SPC and FCT users, respectively. Analysing the two cohorts on SPC vs. FCT, the most frequent comorbidities found during the characterization period were COPD (32.4 vs. 30.8%, *P* < 0.01), diabetes (27.7 vs. 22.5%, *P* < 0.01), and ischaemic heart disease (24.8 vs. 29.2%, *P* < 0.01). The pattern of medications (at least two prescriptions) in SPC vs. FCT was as follows: statins (other than rosuvastatin) 67.3 vs. 47.6% (*P* < 0.001), antihypertensives 75.6 vs. 77.8% (*P* = 0.001), anti-inflammatory and antirheumatic agents 58.3 vs. 53.4% (*P* < 0.001), and antidepressants 16.9 vs. 16.4% (*P* = n.s.). Lastly, the stratification by cardiovascular risk revealed that 32.2% of patients in the SPC cohort and 36.1% of patients in the FCT cohort were classified as very high cardiovascular risk, 51.4 and 49.7%, respectively, as high cardiovascular risk, and 16.4 and 14.2% respectively, as other (low or mild/moderate) cardiovascular risk.

**Table 1:** Baseline demographic and clinical characteristics of patients treated with single-pill combination and free combination treatment, before propensity score matching

	SPC (*n* = 25 886)	FCT (*n* = 7309)	*P*-value
Age, years, mean (± SD)	65.4 (± 11.0)	65.7 (± 11.0)	**<0**.**05**
Male gender, *n* (%)	14 484 (56.0%)	4280 (58.6%)	**<0**.**001**
Comorbidities			
COPD, *n* (%)	8393 (32.4%)	2250 (30.8%)	**<0**.**01**
Ischaemic heart disease, *n* (%)	6431 (24.8%)	2131 (29.2%)	**<0**.**001**
Diabetes, *n* (%)	7159 (27.7%)	1647 (22.5%)	**<0**.**001**
Cerebrovascular disease, *n* (%)	1851 (7.2%)	566 (7.7%)	0.085
Heart failure, *n* (%)	1064 (4.1%)	283 (3.9%)	0.362
Peripheral vascular disease, *n* (%)	791 (3.1%)	273 (3.7%)	**<0**.**01**
Psychiatric disease, *n* (%)	623 (2.4%)	168 (2.3%)	0.592
CKD, *n* (%)	253 (1.0%)	55 (0.8%)	0.077
Co-medications			
Statins^a^ (except rosuvastatin), *n* (%)	17 414 (67.3%)	3478 (47.6%)	**<0**.**001**
Fibrates, *n* (%)	2000 (7.7%)	480 (6.6%)	**0**.**001**
Antihypertensive treatment, *n* (%)	19 576 (75.6%)	5685 (77.8%)	**<0**.**001**
ACE inhibitors, *n* (%)	12 144 (46.9%)	3369 (46.1%)	0.215
Angiotensin II receptor blockers, *n* (%)	10 585 (40.9%)	2961 (40.5%)	0.560
Beta-blockers, *n* (%)	13 787 (53.3%)	4215 (57.7%)	**<0**.**001**
Calcium channel blockers, *n* (%)	7101 (27.4%)	2113 (28.9%)	**<0**.**05**
Antithrombotic agents, *n* (%)	1114 (4.3%)	406 (5.6%)	**<0**.**001**
Antiarrhythmics, *n* (%)	1476 (5.7%)	459 (6.3%)	0.063
Diuretics^b^, *n* (%)	4924 (19.0%)	1376 (18.8%)	0.706
Digoxin, *n* (%)	244 (0.9%)	72 (1.0%)	0.741
Ivabradine, *n* (%)	871 (3.4%)	355 (4.9%)	**<0**.**001**
Anti-inflammatory/antirheumatic products, *n* (%)	15 079 (58.3%)	3903 (53.4%)	**<0**.**001**
Antidepressants, *n* (%)	4370 (16.9%)	1196 (16.4%)	0.295
Cardiovascular risk categories			
Very high risk, *n* (%)	8327 (32.2%)	2640 (36.1%)	**<0**.**001**
High risk, *n* (%)	13 307 (51.4%)	3633 (49.7%)	
Other risk, *n* (%)	4252 (16.4%)	1036 (14.2%)	

Significant *P*-values are in bold.

ACE, angiotensin converting enzyme; CKD, chronic kidney disease; COPD, chronic obstructive pulmonary disease; FCT, free combination treatment; PSM, propensity score matching; SPC, single-pill combination.

^a^Statins prescribed before rosuvastatin.

^b^Diuretics analysed comprised: thiazides, loop diuretics, spironolactone.

Post-PSM, the matched cohorts consisted of 21 927 on SPC and 7309 on FCT (*Table 2*). The two cohorts resulted to be well balanced for demographics, clinical variables, drug prescriptions, and cardiovascular risk categories (for all the parameters, SMD < 0.2). Adherence was evaluated in PSM-balanced cohorts.

**Table 2:** Baseline characteristics of propensity score matching–balanced cohorts of patients treated with single-pill combination and free combination treatment

	SPC (*n* = 21 927)	FCT (*n* = 7309)	*P*-value	SMD
Age, years, mean (± SD)	65.7 (± 10.9)	65.7 (± 11.0)	0.836	0.003
Male gender, *n* (%)	12 852 (58.6%)	4280 (58.6%)	0.934	0.001
COPD, *n* (%)	6701(30.6%)	2250 (30.8%)	0.720	0.005
Psychiatric disease, *n* (%)	525 (2.4%)	168 (2.3%)	0.641	0.006
Anti-inflammatory/antirheumatic products, *n* (%)	11 738 (53.5%)	3903 (53.4%)	0.844	0.003
Antidepressants, *n* (%)	3531 (16.1%)	1196 (16.4%)	0.601	0.007
Cardiovascular risk categories	–	–	0.556	0.015
Very high risk, *n* (%)	7831 (35.7%)	2640 (36.1%)	–	–
High risk, *n* (%)	10 880 (49.6%)	3633 (49.7%)	–	–
Other risk, *n* (%)	3216 (14.7%)	1036 (14.2%)	–	–

COPD, chronic obstructive pulmonary disease; FCT, free combination treatment; PSM, propensity score matching; SMD, standardized mean difference; SPC, single-pill combination.

**Figure 1:**
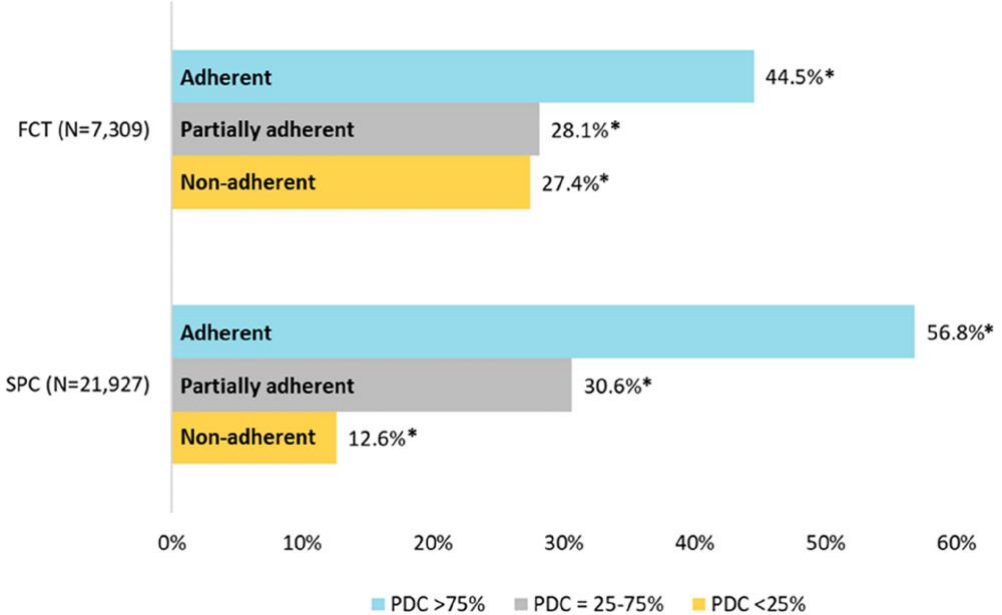
Proportion of adherent, partially adherent, and non-adherent patients in single-pill combination and free combination treatment cohorts, after propensity score matching. *P < 0.001 (single-pill combination vs. free combination treatment).

As shown in *Figure 1*, a significantly higher percentage of patients were adherent (PDC > 75%) to SPC when compared with FCT (56.8 vs. 44.5%, *P* < 0.001). Consistently, a lower percentage of non-adherent (PDC < 25%) patients was found for the SPC vs. FCT (12.6 vs. 27.4%, *P* < 0.001). A slightly but still significant increase of partially adherent patients (PDC = 25–75%) was noticed among SPC vs. FCT (30.6 vs. 28.1%, *P* < 0.001).

**Figure 2:**
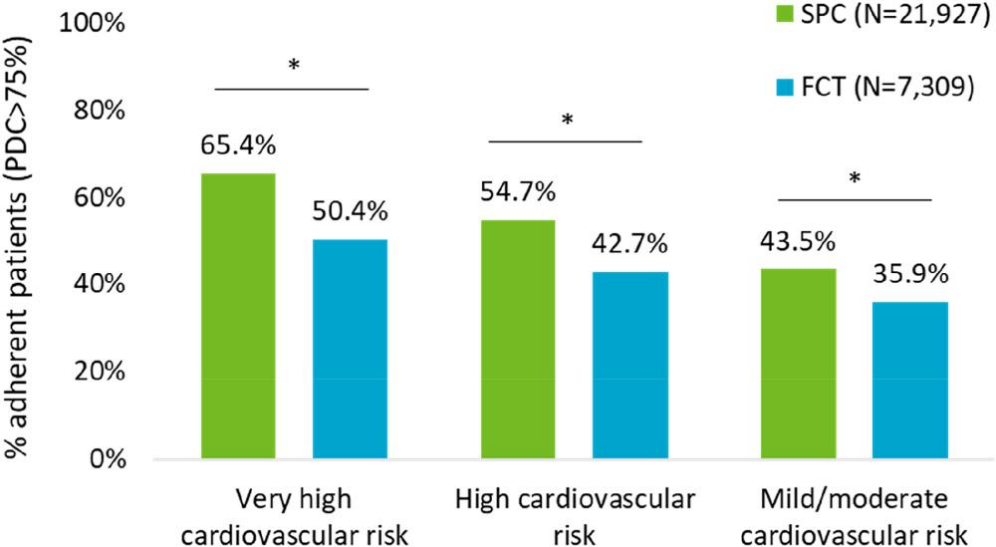
Comparisons between the percentages of adherent patients (proportion of days covered > 75%) receiving single-pill combination vs. free combination treatment in the cardiovascular risk category, after propensity score matching. *P < 0.001 (single-pill combination vs. free combination treatment).

The evaluation of treatment adherence was then replicated in PSM-balanced cohorts after stratifying patients by cardiovascular risk level. Among very high, high, and other cardiovascular risk patients, a significantly higher percentage of adherent patients, by 30, 28, and 21%, respectively, was observed for SPC vs. FCT (very high risk: 65.4 vs. 50.4%, *P* < 0.001; high risk: 54.7 vs. 42.7%, *P* < 0.001; other risk: 43.5 vs. 35.9%, *P* < 0.001) (*Figure 2*).

Achievement of the biochemical lipid target was evaluated at the first year of follow-up in a subset of patients with available laboratory LDL-C measurements at baseline and after 1 year. Among 21 927 patients on SPC, 6063 (28%) had at least one LDL-C test at baseline and 2807 (13%) had at least one LDL-C test at 1- year follow-up. Of 7309 on FCT, 1970 (27%) had at least one LDL-C test at baseline and 1039 (14%) had at least one LDL-C test at 1-year follow-up.

**Figure 3:**
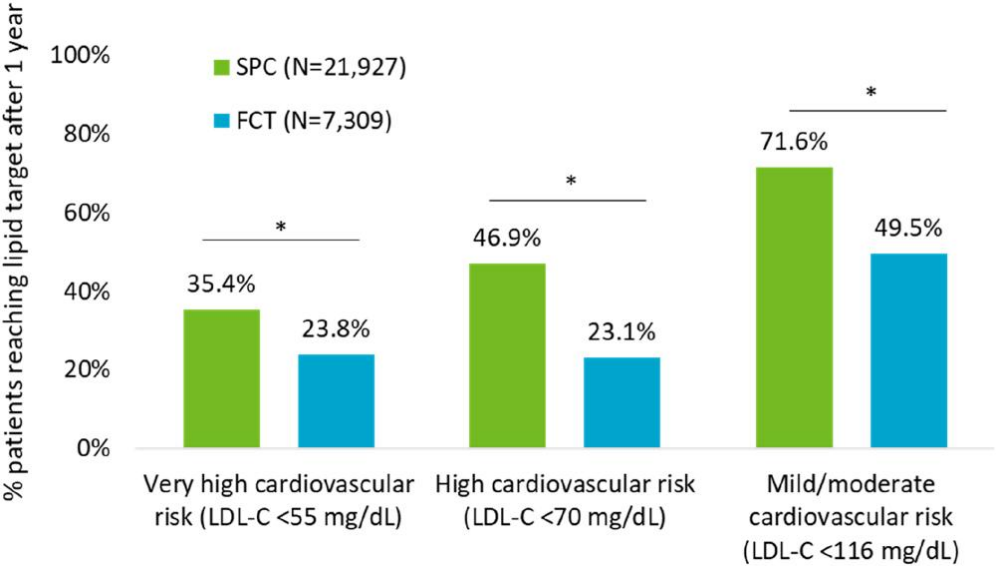
Proportion of patients receiving single-pill combination vs. free combination treatment who reached the LDL cholesterol levels established for each cardiovascular risk category at the first year of follow-up, after propensity score matching. *P < 0.001 (single-pill combination vs. FCT).

The proportion of patients with very high cardiovascular risk reaching LDL-C < 55 mg/dL at 1 year follow-up was higher in SPC vs. FCT (35.4 vs. 23.8%, *P* < 0.001). Similarly, among high-risk patients, 46.9 and 23.1% (*P* < 0.001) of SPC and FCT, respectively, reached LDL-C < 70 mg/dL and among other cardiovascular-risk patients, 71.6 and 49.5% (*P* < 0.001) of SPC and FCT, respectively, reached LDL-C < 116 mg/dL after the first year of follow-up (*Figure 3*).

### Cost analysis

**Figure 4:**
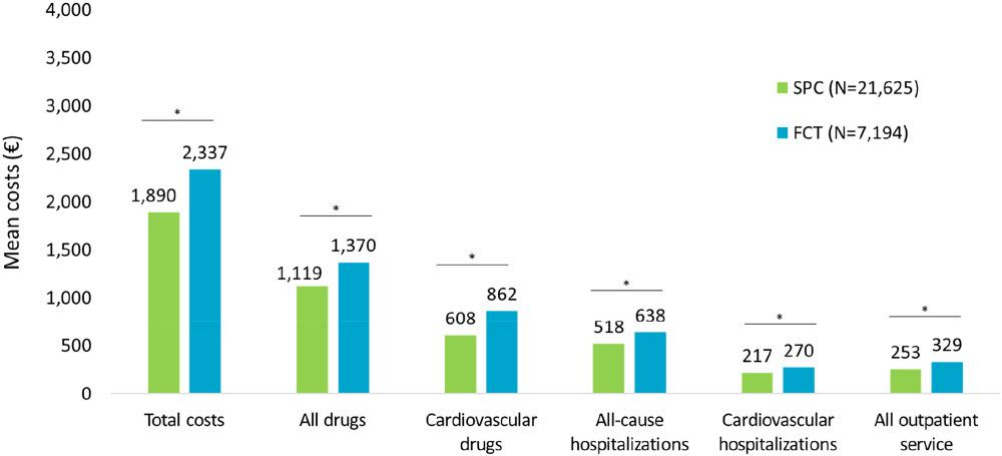
Healthcare costs (outliers excluded) at the first year of follow-up, among patients treated with rosuvastatin/ezetimibe single-pill combination and free combination treatment pill or free combination treatment. *P < 0.001 (single-pill combination vs. free combination treatment).

As shown in *Figure 4*, the total healthcare costs patient at the first year of follow-up were significantly higher in the free combination than in the SPC cohort (2337€ vs. 1890€, *P* < 0.001), mostly due to all drug expenses (1370€ vs. 1119€, *P* < 0.001) and cardiovascular drugs (862€ vs. 608€, *P* < 0.001), followed by all-cause hospitalizations (638€ vs. 518€, *P* < 0.001), cardiovascular-related hospitalizations (270€ vs. 217€, *P* < 0.01), and all outpatient-service costs (329€ vs. 253€, *P* < 0.001).

The generalized linear model conducted on the whole population before PSM-balancing revealed that adherence (PDC > 75%) was associated with an increment of total annual direct costs of 318€ (*P* < 0.001) and FCT therapy with an increment of total annual direct costs of 481€ (*P* < 0.001). Other significant predictors of increased costs were older age (*P* < 0.001), male gender (*P* < 0.001), and therapy with anti-inflammatory and antirheumatic drugs (*P* < 0.001), and antidepressants (*P* < 0.001). Considering the high cardiovascular category as reference, a very high cardiovascular profile resulted in total annual healthcare costs increased of 833€ (*P* < 0.001), while other cardiovascular risk (low or mild/moderate) resulted in costs savings of 472€ (*P* < 0.001) (*Table 3*).

**Table 3:** Generalized linear model for predictors of average total annual direct healthcare costs, before propensity score matching

	€	95% CI		*P*-value
Constant	− 266.49	− 460.94	− 72.04	**<0**.**01**
Age	20.99	18.41	23.57	**<0**.**001**
Male	220.05	164.14	275.95	**<0**.**001**
COPD	358.19	300.41	415.97	**<0**.**001**
Psychiatric disease	165.19	− 9.86	340.24	**0**.**064**
Anti-inflammatory/antirheumatic products	131.64	76.38	186.91	**<0**.**001**
Antidepressants	319.22	246.90	391.55	**<0**.**001**
Cardiovascular risk				
High risk (ref.)	–	–	–	–
Very high risk	833.65	773.59	893.71	**<0**.**001**
Other	− 471.87	− 550.97	− 392.77	**<0**.**001**
Combination				
SPC (ref.)	–	–	–	–
FCT	481.44	417.48	545.41	<0.001
Adherence				
<25% (ref.)	–	–	–	–
25–75%	27.40	−53.88	108.67	0.509
>75%	317.93	242.08	393.79	**<0**.**001**

Significant *P*-values are in bold.

CI, confidence interval; COPD, chronic obstructive pulmonary disease; FCT, free combination treatment; PSM, propensity score matching; SPC, single-pill combination.

## Discussion

The present retrospective observational analysis, carried on a sample population of Italian dyslipidaemic patients, suggested that lipid-lowering therapy with SPC of ROS/EZE increases the chances of being adherent and achieving the recommended LDL-C target levels over FCT across all cardiovascular risk categories. Moreover, from the cost analysis, the single-pill option appeared to be associated with lower healthcare expenditures, essentially due to medications (overall) and hospitalizations.

Data from clinical trials, real-world clinical settings, and systematic literature reviews strongly support the view that, besides statin intensity, combination strategies, and adherence are both key components to a successful lipid-lowering treatment.^30–38^

Regarding the benefits of combination therapies, much evidence has shown that adding ezetimibe to statins rather than doubling the statin dose is a more effective strategy for the achievement of LDL-C goals.^12–15,39^

In our population, the evaluation of patients’ past history of lipid-lowering therapy revealed that in the SPC cohort, there were more patients across a high-risk cardiovascular and other (low or mild/moderate) cardiovascular risk levels. Moreover, among the SPC users, a higher frequency of previous statin use (except rosuvastatin) was noted (67.3%) in comparison with FCT (47.6%). Taken together, these findings seem to suggest an increasing attitude from Italian clinicians in the use of combination therapies based on statins plus ezetimibe in those patients at higher cardiovascular risk, and to choose the SPC option in those who probably failed reaching the lipid goals using statin monotherapies.

The benefits of fixed combinations are currently being supported by increasing literature data, emphasizing how therapy simplification results in improved adherence to lipid-lowering therapy, higher likelihood to C-LDL target attainment which in turn can reduce the risk of atherosclerotic cardiovascular disease^17,34^ and cardiovascular mortality.^35^ Therefore, efforts to ameliorate adherence to statin-based regimens have acquired a crucial importance to optimize their full protective effects. This analysis confirmed that in all cardiovascular risk categories, there was a higher proportion of adherent patients among the group of treatment with SPC regimen of ROS/EZE compared with those with separate pill combinations, independently of cardiovascular risk category. These findings on adherence were mirrored by those on the achievement of lipid targets established by the ESC/EAS Guidelines for each cardiovascular risk category:^5^ there was a higher proportion of patients who reached the LDL-C goals among the group of treatment with SPC regimen of ROS/EZE compared with those receiving an equivalent free combination of separate pills.

Studies have shown that adherence to lipid-lowering therapy is not good in general,^37^ and the mostly reported reasons by patients are concerns about side effects and the attitude to replace statins with non-drug alternative therapies.^39^ Indeed, one critical factor related to suboptimal adherence is the complexity of therapy. It is well known that adherence can significantly benefit from a reduced pill number which allows a simpler self-management of therapy by the patients.^18–27^ The advantage of fixed combinations of statins with ezetimibe lies in the possibility of adding ezetimibe to an existing statin regimen without increasing the number of pills to be taken daily, or therapy complexity.

Consistent with previous evidence,^18–27^ our data, generated from a real-life setting, support the benefits of SPC of statin plus ezetimibe as a valuable approach to improve adherence in patients at high and very high cardiovascular risk. To date, only few analyses investigated in a real-world setting the medication adherence in patients treated with SPC regimens with respect to FCT in Italy. Rea *et al.*^26^ carried out an observational analysis in the Lombardy region that found a markedly better adherence to lipid-lowering therapy in patients prescribed with an SPC of statin/ezetimibe than those who received two separate pills. A recently published work by our group^27^ and the results emerged here corroborated on a national scale the findings by Rea *et al.*,^26^ as we analysed a sample geographically distributed across the country and potentially representative of the entire Italian population.

The inclusion period of this analysis, which ended in June 2020, poses the question of whether COVID-19 could have influenced the results. Hence, with the purpose of assessing the possible interference of the pandemic outbreak on adherence, we replicated the analyses in a subpopulation enrolled before March 2019. The comparisons in the distribution of adherent, partially adherent, and non-adherent patients in SPC vs. FCT cohorts (data not shown) mirrored the results obtained with the main analysis thorough the overall inclusion period. This lacking effect of COVID-19 pandemic on adherence might be explained with the route of delivery of drugs covered by the INHS, including ROS and EZE. The refill of prescriptions by the GP for reimbursed medications takes place in a completely web-based fashion, so that the prescription is electronically transferred from the physician to the community pharmacy. Even during the lock-down period, the patients had the possibility to get their drugs without any contact with the GP, simply going to the nearest territorial pharmacy which had received the electronic filled prescription from the doctor (commonly referred in Italy as ‘paperless’ or ‘dematerialized’ prescription).

To the best of our knowledge, this is the first investigation evaluating the association of the type of formulation of lipid-lowering therapies with healthcare costs in Italy. Here, we noticed that free combinations of ROS/EZE resulted in increased healthcare expenditures compared with the fixed combination for each cost item, namely medications, hospitalizations, and outpatient specialist services. However, the regression analysis showed that adherence was associated with higher costs during follow-up, but this is not surprising, given that better adherence implies larger drug consumptions. Despite this, the SPC option appears anyhow to provide substantial cost savings, feasibly due to the fact that improved adherence can lead to better clinical outcomes and ultimately to a reduction of hospitalization rates, including cardiovascular hospitalizations, use of co-medications or delivery of specialist services. Although up to now, there are no similar cost analyses in the specific setting of statin plus ezetimibe combinations, previous evidence for other cardiovascular-related therapies has corroborated this close link between fixed formulations, improved adherence, and alleviated economic burden.^19,20,28,40,41^ A budget impact model was recently applied by Di Matteo *et al.*^40^ to assess the possible cost savings for the INHS deriving from the use of a fixed-dose combination of acetylsalicylic acid and rosuvastatin. Considering the combination of 100 mg acetylsalicylic acid and with 5, 10, or 20 mg rosuvastatin, for all the statin doses, the SPC option allowed cost savings of €951 201 when using both SPC and FCT or FCT at 50%, and €1902, when using the SPC exclusively, with respect to FCT exclusively.^40^

A similar scenario emerged from various analyses conducted for antihypertensive drugs, which confirmed how patients on fixed formulations displayed better adherence, decreased risk of drug discontinuation, reduced rates of cardiovascular-related hospitalizations, and subsequently lower healthcare costs compared with those on free combinations of the equivalent antihypertensive medications.^19,20^

These data should be interpreted considering some limitations related to the use of administrative databases that might provide lacking or partial clinical documentation on comorbidities and other confounders, with a potential impact on the final results. In this context, one main limitation lies in the criteria used to stratify our population by cardiovascular risk with reference to those from the ESC/EAS 2019 guidelines.^5^ Since the administrative databases are meant to collect data for reimbursement purposes, the cardiovascular risk factors could be only identified using ICD-9-CM codes for hospitalizations and ATC codes for drug prescriptions as proxy of diagnosis. However, the margin of error in cardiovascular risk stratification due to this approach should not be detrimental to the findings, which were comparable across all the cardiovascular risk categories.

Data on pharmacological treatments (drug adherence) were collected from medical prescription and dispensing information, thus the reasons behind non-adherence were not captured. Moreover, adherence was not evaluated as a continuous variable, but by means of the frequency of patients adherent, partially adherent and non-adherent, based on PDC thresholds. Besides, we could not discriminate between the different rosuvastatin daily dosages commonly prescribed, 5, 10, and 20 mg. The comparative analysis between the two cohorts was performed by balancing them with a PSM approach: although PSM represents a valuable methodology to abate non-randomization bias in observational analyses, it was based on the covariates extracted from the databases and evaluated at baseline, thus some factors not traceable from the administrative flows were not included and it is not possible to assess the role of these unknown/unmeasured confounders. Moreover, the design followed the intention to treat approach. Finally, the retrospective nature of the study and the short follow-up prevent us from extending our conclusions to long-term cardiovascular outcomes.

Despite these limitations, the main strength of our analysis is the large sample representativeness, as we accessed the administrative database of healthcare bodies geographically distributed across North, central, and South Italy covering ∼11% of the national population. Furthermore, the reliability of the data emerging from the present analysis appears to be supported by a substantial consistency with evidence from other countries.

## Conclusion

This analysis carried out in a setting of Italian clinical practice provides real-world evidence on the achievement of biochemical outcomes and treatment adherence of patients to ROS/EZE in SPC or FCT. The results showed that treatment with ROS/EZE SPC was associated with better medication adherence, greater likelihood of LDL-C target achievement, and reduced healthcare costs compared with FCT, regardless of cardiovascular risk level.

## Lead author biography


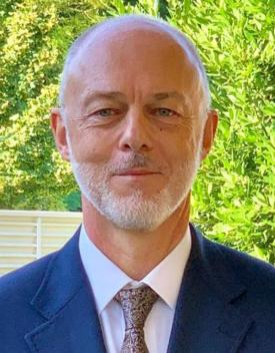


Prof. Alberto Zambon received an MD degree (1988), is board certified in Endocrinology and Metabolism (1993), and completed PhD in Gerontology (1998) at the Padua University. He was a Post-Doctoral Fellow at the Endocrinology, Metabolism and Nutrition Division, School of Medicine, University of Washington, Seattle, appointed as an Acting Assistant Professor of Medicine, and holds a position of Affiliate Assistant Professor of Medicine. He was the author of 118 peer-reviewed articles (*h-*index 41). Currently, he is an Executive Committee member of the EAS, Gold Heart Member of the AHA and FAHA.

## Supplementary Material

oeae074_Supplementary_Data

## Data Availability

All data used for the current study are available upon reasonable request from CliCon which is the body entitled of data treatment and analysis by all the Local Health Units involved.
