# Supplementary material for: A real-world analysis of adherence, biochemical outcomes, and healthcare costs in patients treated with rosuvastatin/ezetimibe as single-pill combination vs. free combination in Italy
Source: Eur Heart J Open. 2024 Aug 28;4(5):oeae074. doi: 10.1093/ehjopen/oeae074 (PMC11416014; doi:10.1093/ehjopen/oeae074)
Supplement: oeae074_Supplementary_Data [file oeae074_supplementary_data.docx]

**Criteria for stratifying patients by cardiovascular risk**

Patients were stratified into the following groups:

- **Very high cardiovascular risk:** Patients with a diagnosis of diabetes mellitus (verified by at least two prescriptions for ATC drugs: A10 or ICD-9-CM: 250), with a diagnosis of a previous cardiovascular event (ICD-9-CM: 410-414, 440, 443), cerebrovascular event (ICD-9-CM: 430-438), chronic kidney disease (ICD-9-CM: 585), or percutaneous transluminal coronary angioplasty (ICD-9-CM: V4582, 0066, 3609, 3610).
- **High cardiovascular risk:** Patients with at least two prescriptions for antihypertensive drugs (ATC: C03, C07, C08, C09) or cardiac therapy drugs (ATC: C01) or antiplatelet agents (ATC: B01AC) or anticoagulants (ATC: B01AA, B01AB).
- **Other cardiovascular risk:** Patients not meeting any of the above conditions.
